# Vitamin K antagonist vs direct oral anticoagulants with antiplatelet therapy in dual or triple therapy after percutaneous coronary intervention or acute coronary syndrome in atrial fibrillation: Meta‐analysis of randomized controlled trials

**DOI:** 10.1002/clc.23224

**Published:** 2019-07-09

**Authors:** Vincent Roule, Pierre Ardouin, Clément Briet, Adrien Lemaitre, Mathieu Bignon, Rémi Sabatier, Laure Champ‐Rigot, Paul Milliez, Katrien Blanchart, Farzin Beygui

**Affiliations:** ^1^ CHU de Caen Normandie, Service de Cardiologie Caen France; ^2^ Normandie Univ, UNICAEN, EA 4650 Signalisation, électrophysiologie et imagerie des lésions d'ischémie‐reperfusion myocardique Caen France; ^3^ ACTION Academic Group Pitié Salpêtrière University Hospital Paris France

**Keywords:** atrial fibrillation, direct oral anticoagulant, dual therapy, percutaneous coronary intervention, triple therapy

## Abstract

**Background:**

The combination of vitamin K antagonists (VKA) for atrial fibrillation (AF) and antiplatelet agents following percutaneous coronary intervention (PCI) is associated with an increased bleeding risk.

**Hypothesis:**

Direct oral anticoagulants (DOAC) are associated with a greater safety profile but the optimal antithrombotic treatment strategy, especially when considering ischemic events, is unclear.

**Methods:**

We performed a meta‐analysis of randomized controlled trials comparing outcomes in AF patients following PCI and/or acute coronary syndrome (ACS) when treated with DOAC vs VKA, both in combination with one (dual) or two (triple) antiplatelet regimens. A systematic review was performed by searches of electronic databases MEDLINE (source PubMed) and the Cochrane Controlled Clinical Trials Register Database as well as Cardiology annual meetings. Three studies were finally included.

**Results:**

Compared to VKA triple therapy, the use of DOAC was associated with a decreased risk of any bleeding (relative risk [RR] 0.68 [0.62; 0.74]), major bleeding (RR 0.61 [0.51; 0.75]) and intracranial bleeding (RR 0.33 [0.17; 0.66]) and similar rates of the composite efficacy endpoint (RR 1.0 [0.87; 1.14]) and its components. Similar and consistent results were observed with both dual and triple therapy including a DOAC compared to VKA.

**Conclusion:**

Our meta‐analysis supports the use of dual therapy combining a DOAC and clopidogrel as the default regimen in most AF patients after PCI and/or ACS.

## 1 INTRODUCTION

Atrial fibrillation (AF) has been reported in 3% to 12% of patients undergoing percutaneous coronary intervention (PCI).^1^, ^2^, ^3^ The combination of vitamin K antagonists (VKA) with aspirin and clopidogrel—called “triple therapy”—was associated with an increased risk of major bleeding up to 2.2% within the first month and 12% at 1 year in acute coronary syndromes (ACS) patients.^4^, ^5^

An alternative to triple therapy is the use of dual therapy associating VKA and clopidogrel alone assessed in the setting of all‐coming PCI patients who required anticoagulation.^6^ Dual therapy was associated with a significantly lower rate of bleeding at 1 year. On the other hand, direct oral anticoagulants (DOAC) were reported to be similarly efficient as warfarin in the prevention of stroke in patients with AF and associated with lower rates of major or intracranial bleeding.^7^, ^8^, ^9^, ^10^ The greater safety profile of DOAC associated with antiplatelet agents over triple therapy with VKA was confirmed in two recent randomized controlled trials (RCT) in patients with AF treated with PCI.^11^, ^12^ Neither trial was designed to assess whether greater safety profile was due to the use of the DOAC or to the removal of aspirin therapy. Even if rates of major adverse cardiac events (MACE) were not different between study arms, both studies were underpowered for the comparison of DOAC and VKA with respect to ischemic outcomes. Some clinicians still fear to stop aspirin after PCI and/or ACS in patient treated with DOAC. The optimal antithrombotic treatment strategy especially when considering ischemic events remains unclear.

We conducted a systematic review and meta‐analysis of RCTs to compare the safety and efficacy of DOAC vs VKA use either in a dual or triple combination therapy in AF patients requiring such combination in the setting of PCI and/or ACS.

## 2 METHODS

### 2.1 Study selection

We followed the Preferred Reporting Items for Systematic Reviews and Meta‐Analysis (PRISMA) guidelines for the systematic review and meta‐analysis (see PRISMA checklist in Appendix S1). We conducted a systematic literature review by formal searches of the electronic databases MEDLINE (source PubMed) and the Cochrane Controlled Clinical Trials Register Database as well as European Society of Cardiology, American Heart Association, and American College of Cardiology annual meetings through March 18, 2018. The following search terms were used: “vitamin K antagonist” OR “VKA” OR “warfarin” OR “dabigatran” OR “rivaroxaban” OR “apixaban” OR “edoxaban” OR “novel oral anticoagulant” OR “new oral anticoagulant” OR “NOAC” OR “direct oral anticoagulant” OR “DOAC” AND “percutaneous coronary intervention” OR “PCI” OR “coronary stent implantation” OR “acute coronary syndrome” AND “atrial fibrillation” OR “AF” AND “randomized controlled trial” OR “randomized” OR “trials.” References from reviews and selected articles were also reviewed for potential relevant citations. Studies were searched for and evaluated by two independent reviewers (VR and PA).

We restricted our analysis to the trials that met all of the following inclusion criteria: (a) randomized controlled comparison between DOAC and VKA, (b) in patients with AF following PCI and/or ACS, (c) available outcomes involving main adverse cardiac and cerebrovascular events (MACCEs) and major bleeding, and (d) English‐language publications.

### 2.2 Outcome definition

Any bleeding ranging from minor to severe and major bleeding were the safety endpoints. Major bleeding complications were reported as defined in each study. The efficacy endpoints were all‐causes death, cardiovascular death, myocardial infarction, stent thrombosis, stroke, and the composite efficacy endpoint (MACCEs) as defined in each study. Outcomes were based on the longest follow‐up available for each study.

### 2.3 Statistical analysis

The total numbers of patients experiencing or not the outcomes of interest in each arm extracted directly from the publications were used for the analyses. Results are presented as relative risks (RR) with 95% confidence intervals (95% CI). Outcomes from individual studies were combined using the Mantel‐Haenzel fixed and random‐effect models. Heterogeneity across studies was studied by the Cochran's Q statistic with a *P*‐value set at .1. The *I* ^2^ was also taken into account regardless of the *P*‐value. An *I* ^2^ of ≥50% was pre‐specified as the threshold considered too high to provide consistent analysis. The random‐effect model was considered for the analysis. Tests were two‐tailed and a *P*‐value of <.05 was considered statistically significant. Funnel plots were used to assess publication bias. As AUGUSTUS included patients with ACS but no PCI, we did a complementary analysis after excluding such patients. We also analyzed different subgroups of DOAC patients (dual or triple therapy, low or high dose) in comparison with VKA regimens. In an exploratory analysis, we also compared data between subgroups of DOAC: dual therapy vs triple therapy, low dose vs high dose in studies providing such data. R software version 3.0.0 (April 3, 2013) for MacOS (R Foundation for Statistical Computing) with Meta package was used for the statistical analysis. The methodological quality of the randomized trials was assessed by Cochrane's Collaboration tool for assessing risk of bias.

## 3 RESULTS

**Figure 1:**
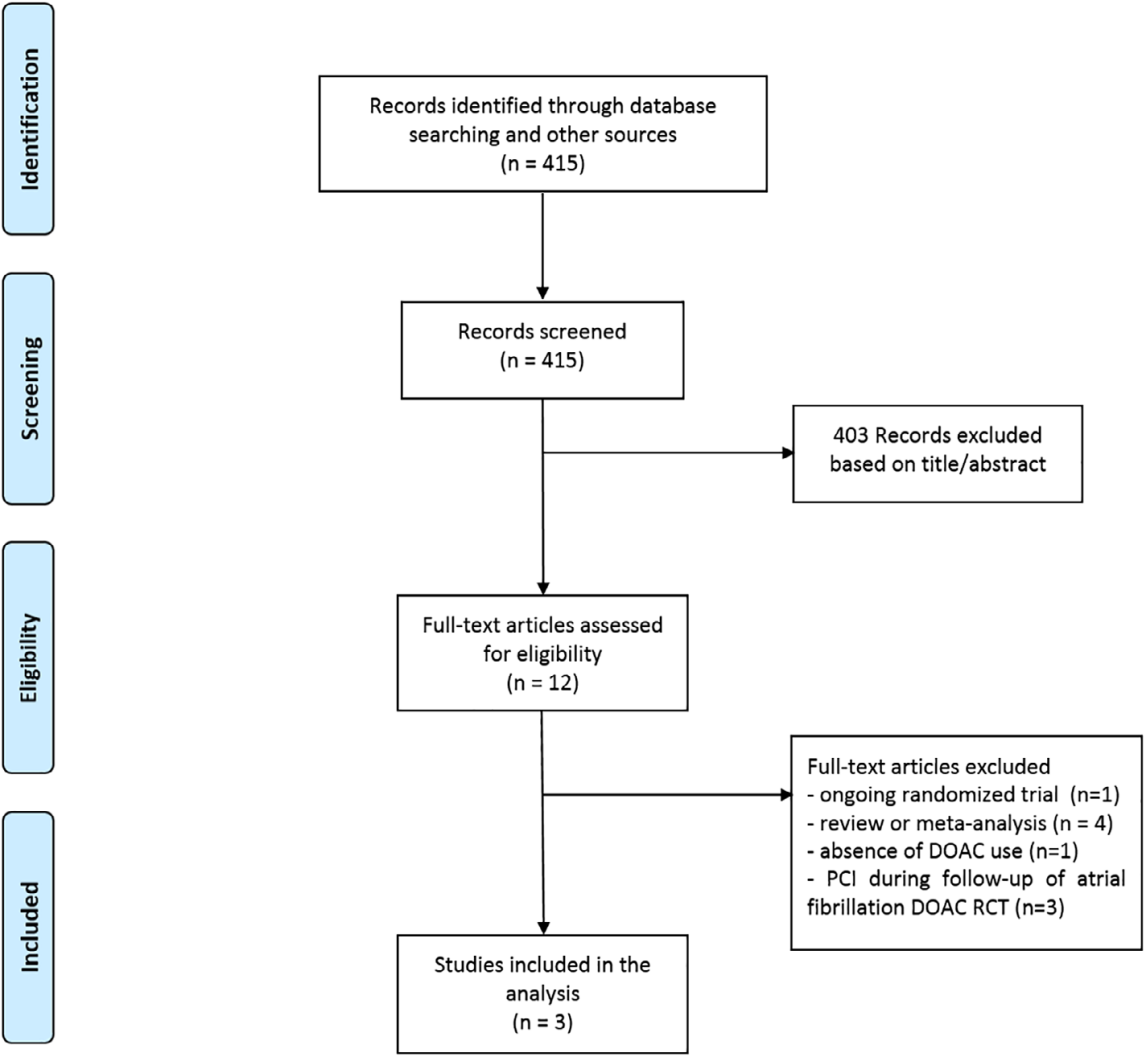
Flow diagram of meta‐analysis trial selection. DOAC, direct oral anticoagulants; PCI, percutaneous coronary intervention; RCT, randomized controlled trials

Three RCT^11^, ^12^, ^13^ representing 9463 patients were selected for the meta‐analysis. The review and selection process is depicted in Figure 1. The endpoints were collected at 12 months in PIONEER AF‐PCI, at 14 months in RE‐DUAL PCI and at 6 months in AUGUSTUS study. The major characteristics of the patients of each study are detailed in Table 1.

**Table 1:** Baseline characteristics of the patients in the included studies

	PIONEER AF‐PCI			RE‐DUAL PCI			AUGUSTUS	
	Dual therapy with rivaroxaban 15 mg (n = 709)	Triple therapy with rivaroxaban 2.5 mg^a^ (n = 709)	Triple therapy with VKA (n = 706)	Dual therapy with Dabigatran 110 mg^a^ (n = 981)	Dual therapy with Dabigatran 150 mg^a^ (n = 763)	Triple therapy with VKA (n = 981)	Apixaban (n = 2306)	VKA (n = 2308)
Age (year)	70.4 ± 9.1	70 ± 9.1	69.9 ± 8.7	71.5 ± 8.9	68.6 ± 7.7	71.7 ± 8.9	70.4^b^	70.9^b^
Female sex	181 (25.5)	174 (24.5)	188 (26.6)	253 (25.8)	171 (22.4)	231 (23.5)	670 (29.1)	667 (28.9)
Diabetes	204 (28.8)	199 (28.1)	221 (31.3)	362 (36.9)	260 (34.1)	371 (37.9)	842 (36.5)	836 (36.2)
Hypertension	520 (73.3)	519 (73.2)	532 (75.4)	na	na	na	2042 (88.6)	2031 (88.0)
Previous stroke	0	0	0	74 (7.5)	52 (6.8)	100 (10.2)	326 (14.2)	307 (13.4)
Creatinine clearance (mL/min)	78.3 ± 31.3	77.5 ± 31.8	80.7 ± 30.0	76.3 ± 28.9	83.7 ± 31.0	75.4 ± 29.1	na	na
ACS as index event	361 (51.5)	374 (53.2)	361 (52.2)	509 (51.9)	391 (51.2)	475 (48.4)	1420 (61.8)	1391 (60.5)
Drug‐eluting stent	464 (65.4)	471 (66.8)	468 (66.5)	804 (82.1)	621 (81.5)	826 (84.6)	na	na

Abbreviations: ACS, acute coronary syndrome; AF, atrial fibrillation; PCI, percutaneous coronary intervention; VKA, vitamin K antagonist.

a Twice daily.

b Median.

### 3.1 Safety endpoints

Major bleeding complications were defined according to the Thrombolysis in Myocardial Infarction (TIMI) hemorrhage classification in PIONEER AF‐PCI trial^12^ and the International Society on Thrombosis and Hemostasis (ISTH) in RE‐DUAL PCI^11^ and AUGUSTUS trials.^13^

**Figure 2:**
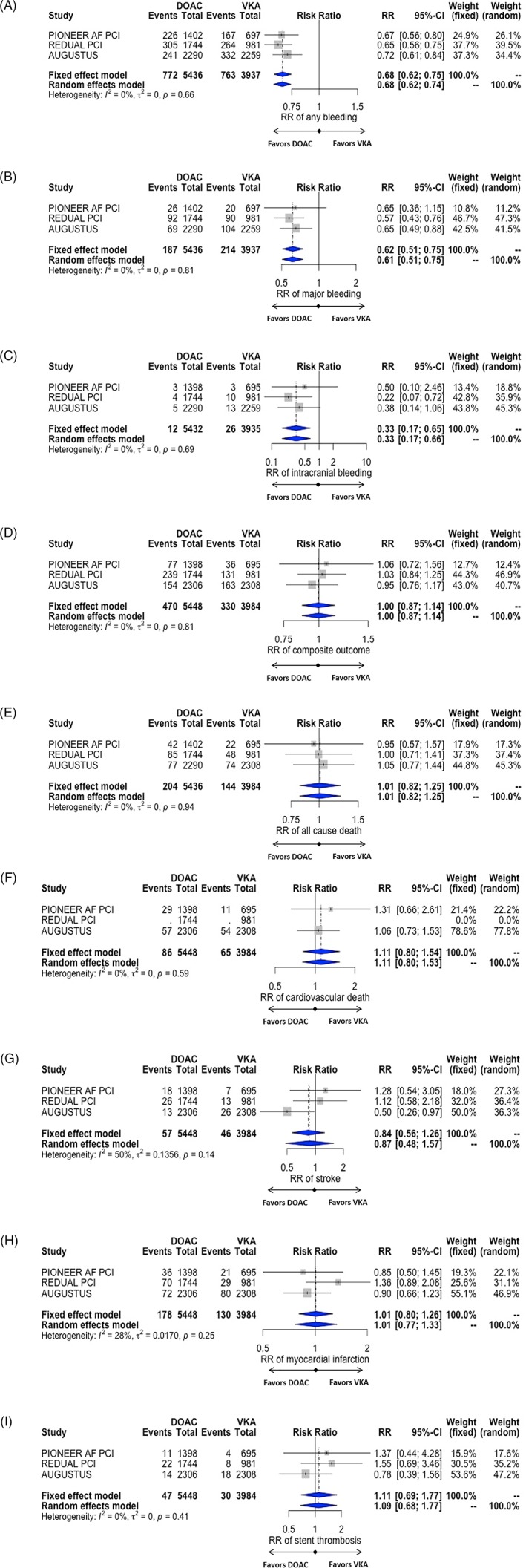
Forest plots of selected studies comparing the effect of direct oral anticoagulants (DOAC) vs vitamin K antagonists (VKA) on any significant bleeding (A), major bleeding (B), intracranial bleeding (C), the composite efficacy endpoint of the studies (D), all‐causes death (E), cardiovascular death (F), stroke (G), myocardial infarction (H) and stent thrombosis (I). RR, risk ratio

Our meta‐analysis showed with very high consistency (*I* ^2^ = 0%) that patients treated with DOAC were at lower risk of any significant bleeding (RR 0.68 [0.62; 0.74]; Figure 2A), major bleeding (RR 0.61 [0.51; 0.75]; Figure 2B) and intracranial bleeding (RR 0.33 [0.17; 0.66]; Figure 2C) compared to those receiving triple therapy with VKA. The magnitude of risk reduction was consistently highest in patients treated with DOAC in a dual therapy strategy for any significant bleeding (RR 0.65 [0.59; 0.73]), major bleeding (RR 0.57 [0.46; 0.72]), and intracranial bleeding (RR 0.24 [0.09; 0.68]; see Figure S2) compared to corresponding VKA arms. Safety results remained similar in comparison with VKA arms when considering only PCI patients in the AUGUSTUS trial (Figure S1), as well as in analyses of subgroups defined by triple or dual therapy (Figures S2‐S3) and by low or high DOAC doses (Figures S4‐S5).

The exploratory analysis comparing different subsets of DOAC‐regimens did not find any significant differences. However, trends toward lower bleeding risks (RR 0.71 [0.40;1.25], *I* ^2^ = 91% for any significant bleeding and RR 0.72 [0.30; 1.73], *I* ^2^ = 74% for major bleeding) were found in association with dual compared to triple therapy with DOAC (Figure S6) and lower compared to higher DOAC doses (RR 0.89 [0.65;1.23], *I* ^2^ = 76% for any significant bleeding and RR 0.88 [0.62;1.25], *I* ^2^ = 0% for major bleeding; Figure S7). When considering each DOAC subgroup of the studies, we found a lower risk of any significant bleeding (RR 0.68 [0.62; 0.75]) and major bleeding (RR 0.63 [0.53; 0.75]; Figure 2B) compared to VKA (Figure S8).

### 3.2 Efficacy endpoints

Patients treated with DOAC had similar risk of the composite efficacy endpoint (RR 1.0 [0.87; 1.14]; Figure 2D), all‐cause death (RR 1.01 [0.82; 1.25]; Figure 2E), cardiovascular death (RR 1.11 [0.80; 1.53]; Figure 2F), stroke (RR 0.87 [0.48; 1.57]; Figure 2G), myocardial infarction (RR 1.01 [0.77; 1.33]; Figure 2H), and stent thrombosis (RR 1.09 [0.68; 1.77]; Figure 2I) compared to those receiving triple therapy with VKA. The analyses were highly consistent except for stroke (*I* ^2^ = 50%) where a significant reduction of stroke was observed only in the AUGUSTUS trial in association with apixaban.^13^ Efficacy results remained similar in comparison with VKA arms when considering PCI patients in the AUGUSTUS trial (Figure S1), as well as subgroups defined by triple or dual therapy (Figure S2‐S3). Non‐significant trends were found toward higher ischemic events risks in association with lower (RR 1.11 [0.91;1.35], *I* ^2^ = 0%; Figure S4) but not higher (RR 0.98 [0.78; 1.23], *I* ^2^ = 0%; Figure S5) DOAC doses compared to VKA.

The exploratory analysis comparing different subsets of DOAC‐regimens did not find any significant differences. However, trends toward higher ischemic risks were found in association with lower compared to higher DOAC doses (RR 1.10 [0.75;1.61], *I* ^2^ = 59%; Figure S7) while such trends were not found when comparing dual vs triple therapy with DOAC (RR 1.06 [0.82; 1.37], *I* ^2^ = 0%; Figure S6). Efficacy results remained similar in comparison with VKA when considering each DOAC subgroup of the studies (RR 1.02 [0.90; 1.16]; Figure S8).

Funnel plots showed no publications bias (Figure S9). All the trials were judged to be at low risk of bias via the Cochran's Collaboration tool for risk assessment (Table S1). Table S2 resumed the design and characteristics of the studies.

## 4 DISCUSSION

Our meta‐analysis shows, with strong consistency between studies, that in AF patients treated with PCI/ACS, the association of DOAC and antiplatelet agents was associated with a significant decrease in the risks of any, major and intracranial bleeding with similar rates of efficacy endpoints as compared to VKA‐based regimen. The most important reduction of bleeding risk was observed with dual therapy with DOAC which was associated with similar efficacy as compared to VKA.

The major risk of combining oral anticoagulation and antiplatelet therapy in patients with AF treated with PCI is bleeding. There is a high, early, and long‐term, risk of bleeding with the triple therapy including VKA,^5^, ^14^, ^15^ with a 2‐ to 3‐fold increase of bleeding complications compared to VKA. Such events are associated with a short‐ and long‐term higher risk of mortality.^16^, ^17^ A study reported that half of the patients experiencing major bleeding with triple therapy died within 6 months; all of which as a consequence of intracranial bleeding.^18^ The lower risk of major bleeding associated with dual therapy including VKA^6^ and further with DOAC^12^ appears as a major improvement of care in such patients. Our study showed that DOAC use reduces major bleeding by 39% and intracranial bleeding by 67% compared to VKA. When DOAC were used as dual therapy with a single antiplatelet agent, major bleeding and intracranial bleeding were reduced by 43% and 76%, respectively. Considering the stepwise increase of the risk of death associated with increased severity of bleeding,^19^ the consistent safety profile of DOAC showed in our analysis supports their use as the default strategy especially in a dual therapy regimen.

While RCT studying the association of DOAC and antiplatelet agents in AF patients undergoing PCI consistently showed a greater safety profile of DOAC over VKA, none was powered to assess efficacy thrombotic endpoints. Patients with AF are at higher risk of thromboembolic events compared to those without AF. The risk of myocardial infarction in such patients is also about twice higher^20^ and increases with the use of PCI^21^ as compared to those without AF. Our pooled meta‐analysis supports comparable efficacy of DOAC vs VKA in this setting. Additionally, dual therapy using a DOAC and one antiplatelet agent—mostly clopidogrel—was shown to be safer than and as effective as dual or triple therapy using VKA. There rates of stent thrombosis were similar between studied arms and very low in the included studies, possibly because of the use of newer generation drug‐eluting‐stent and the limited proportion of PCI for ST‐elevation myocardial infarction.^22^, ^23^ Such characteristics may have blunted a difference between DOAC and the possibly more potent anticoagulation by VKA. On the other hand, major bleeding associated with VKA and triple therapy may lead to antithrombotic treatment interruption and in turn promote ischemic events.^24^ Bleeding can reduce oxygen delivery to the myocardium and promote platelet activation.^25^ Major bleeding is associated with an increased risk of recurrent ischemic events including myocardial infarction and stroke in ACS patients.^17^, ^19^ Consequently, by reducing bleeding complications, the use of DOAC may have led to reduced ischemic events too. The same consideration applies to dual therapy which showed comparable thromboembolic events rates. This is in concordance with results of a large registry and a previous meta‐analysis that include dual therapy with VKA.^26^, ^27^ Unlike other studies included in our analysis, there was an important 50% reduction of the risk of stroke with apixaban compared to VKA in the AUGUSTUS trial.^13^ This may be explained by the use of the approved dose of apixaban tested in the pivotal trial for stroke prevention,^9^ unlike the doses of rivaroxaban (15 or 5 mg per day) and dabigatran 110 mg used in PIONEER AF‐PCI and RE‐DUAL PCI trials, respectively.^11^, ^12^ In absence of a head to head comparison, the relative efficacy of DOAC on the prevention of stroke is not known. Hence, a dose effect may explain the heterogeneity found in our analysis for stroke. Concordantly our subgroup analysis and exploratory analysis comparing lower vs higher doses of rivaroxaban and dabigatran, showed a trend toward higher risks of ischemic events including stroke and stent thrombosis in association with lower doses. Hence, our analysis not only supports the preferential use of approved doses of apixaban in AF patients following PCI or ACS as assessed in the AUGUSTUS trial, but also higher doses of rivaroxaban (15 mg) and dabigatran (150 mg) in absence of high bleeding risk.

International guidelines already recommend the preferential use of DOAC over VKA in AF patients treated with PCI. However while European guidelines^28^, ^29^ recommend triple therapy as the default strategy except for patients at very high bleeding risk, the recent AHA/ACC/HRS guidelines^30^ recommend that dual therapy (including clopidogrel with rivaroxaban 15 mg or dabigatran 150 mg twice daily or dose‐adjusted VKA) should be considered for most patients. Our study supports the overall preferential use of DOAC as well as a dual therapy strategy for most patients in this setting as dual therapy was associated with the strongest risk reduction for bleeding without higher ischemic risk. The rates of significant bleeding are highest within the first 30 days after PCI and twice higher than those of ischemic events.^11^, ^12^, ^13^ Hence, by shifting the choice of the antithrombotic regimen toward dual therapy with a DOAC in most patients immediately following PCI, a significant reduction of bleeding without an excess in thrombotic risk may be anticipated.

### 4.1 Limits

Our meta‐analysis was not performed on individual patients' data. Hence, analyses could not take in to account the individual levels of risk. A limited proportion of included patients presented with ST‐elevation myocardial infarction. Knowing the higher risk of stent thrombosis associated with the latter condition, caution should be taken to extend the results in this setting. The doses of rivaroxaban used in the PIONEER AF‐PCI study^12^ were lower than those used for stroke prevention in the ROCKET‐AF trial.^10^ Although the results of PIONEER trial are in line with other trials included in our analysis, the effect of a 20 mg recommended dose remains un‐assessed. The comparison between low and high doses of DOAC and between dual and triple therapy using DOAC are only exploratory as with the exception of AUGUSTUS trial^13^ the included trials were not designed to assess antiplatelet regimens individually. Our research was limited to two main databases for studies retrieval. Substantial heterogeneity exists in between trials in terms of trial design as well as type and duration of antiplatelet/antithrombotic therapy used, which could affect interpretation of our results. Finally, only three randomized studies were included in the analysis which might limit the assessment of publication bias.

## 5 CONCLUSION

Our study showed that the association of DOAC and antiplatelet agents after PCI and/or ACS in AF patients was associated with a lower risk of major bleeding, especially when considering dual therapy, while preventing thrombotic events similarly to VKA‐based regimens. Because of low rates of bleeding and no increase in risk of thrombotic events, dual therapy combining a DOAC and clopidogrel appears as the default regimen in most patients in this setting.

## CONFLICT OF INTEREST

The authors declare no potential conflict of interests.

## Supporting information

FIGURE S1 Meta‐analysis with percutaneous coronary intervention (PCI) only patients from AUGUSTUS
FIGURE S2: Comparison between dual therapy with direct oral anticoagulants (DOAC) and corresponding vitamin K antagonists(VKA) group (triple therapy VKA in PIONEER AF‐PCI and RE‐DUAL PCI, dual therapy with VKA in AUGUSTUS)
FIGURE S3: Comparison between triple therapy with DOAC and triple therapy with VKA
FIGURE S4: Comparison between low doses of DOAC and VKA
FIGURE S5: Comparison between high doses of DOAC and VKA
FIGURE S6: Comparison between dual therapy and triple therapy with DOAC
FIGURE S7: Comparison between low dose and high dose of DOAC
FIGURE S8: Comparison between each DOAC subgroup (according studies) and VKA. PS: no subgroup data were available for AUGUSTUS trial according the dose of Apixaban.
FIGURE S9: Funnel plots
TABLE S1 Risk of bias in randomized studies, based on the Cochrane Risk of Bias Tool for Randomized Controlled Trials
TABLE S2 Design and characteristics of the selected studies.

**APPENDIX S1** PRISMA 2009 checklist
