# Supplementary material for: Vitamin K antagonist vs direct oral anticoagulants with antiplatelet therapy in dual or triple therapy after percutaneous coronary intervention or acute coronary syndrome in atrial fibrillation: Meta‐analysis of randomized controlled trials
Source: Clin Cardiol. 2019 Jul 9;42(9):839–46. doi: 10.1002/clc.23224 (PMC6727878; doi:10.1002/clc.23224)
Supplement: Supplementary file 1 — FIGURE S1 Meta‐analysis with percutaneous coronary intervention (PCI) only patients from AUGUSTUS FIGURE S2: Comparison between dual therapy with direct oral anticoagulants (DOAC) and corresponding vitamin K antagonists(VKA) group (triple therapy VKA in PIONEER AF‐PCI and RE‐DUAL PCI, dual therapy with VKA in AUGUSTUS) FIGURE S3: Comparison between triple therapy with DOAC and triple therapy with VKA FIGURE S4: Comparison between low doses of DOAC and VKA FIGURE S5: Comparison between high doses of DOAC and VKA FIGURE S6: Comparison between dual therapy and triple therapy with DOAC FIGURE S7: Comparison between low dose and high dose of DOAC FIGURE S8: Comparison between each DOAC subgroup (according studies) and VKA. PS: no subgroup data were available for AUGUSTUS trial according the dose of Apixaban. FIGURE S9: Funnel plots TABLE S1 Risk of bias in randomized studies, based on the Cochrane Risk of Bias Tool for Randomized Controlled Trials TABLE S2 Design and characteristics of the selected studies. [file CLC-42-839-s001.docx]

**Supplementary Appendix**

**Figure S1: meta-analysis with PCI only patients from AUGUSTUS**

**S1-A Any significant bleeding**


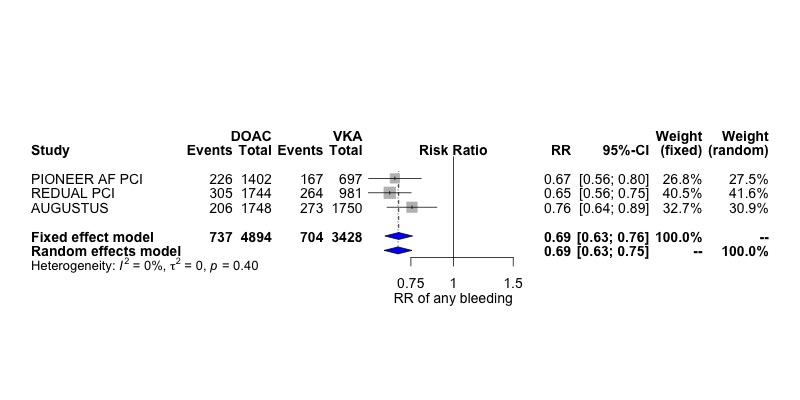


**Favors VKA**

**Favors DOAC**

**S1-B Composite efficacy endpoint**


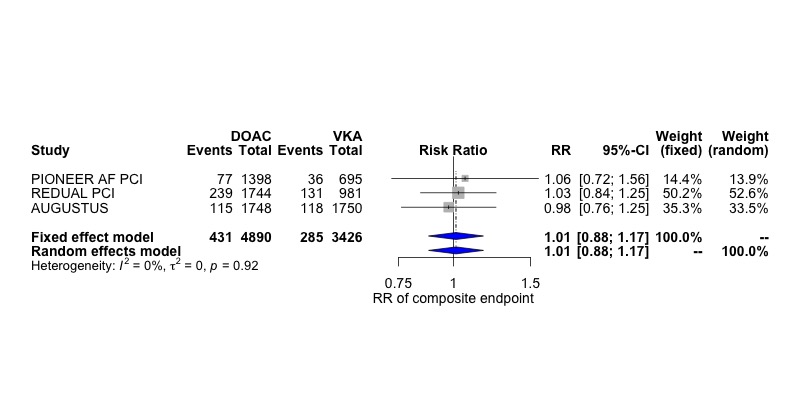


**Favors VKA**

**Favors DOAC**

**Figure S2:** **Comparison between dual therapy with DOAC and corresponding VKA group (triple therapy VKA in PIONEER AF-PCI and RE-DUAL PCI, dual therapy with VKA in AUGUSTUS)**

**S2-A Any significant bleeding**

**
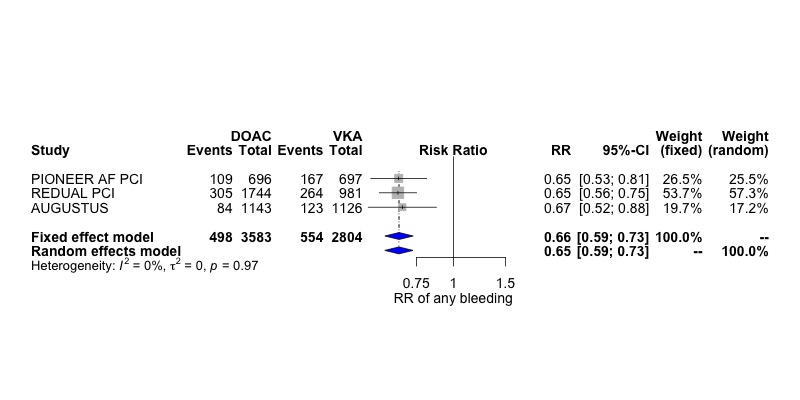
**

**Favors VKA**

**Favors DOAC**

**S2-B Major bleeding**

**
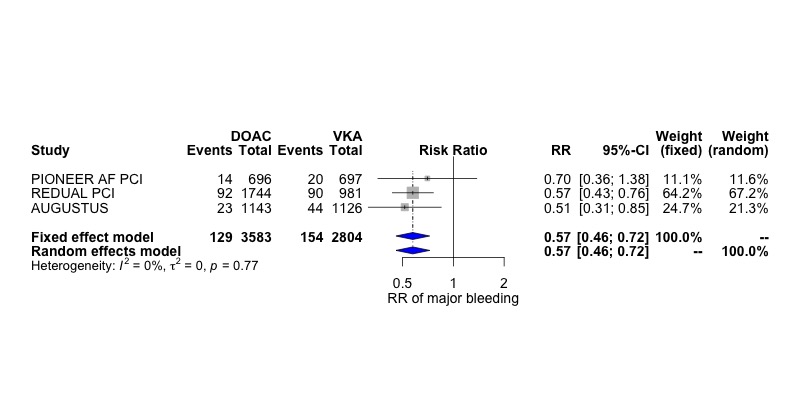
**

**Favors VKA**

**Favors DOAC**

**S2-C Intracranial bleeding**

**
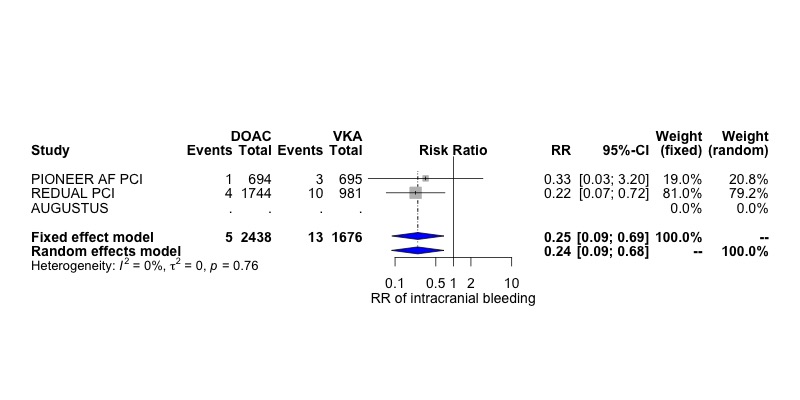
**

**Favors VKA**

**Favors DOAC**

**S2-D Composite efficacy endpoint**

**
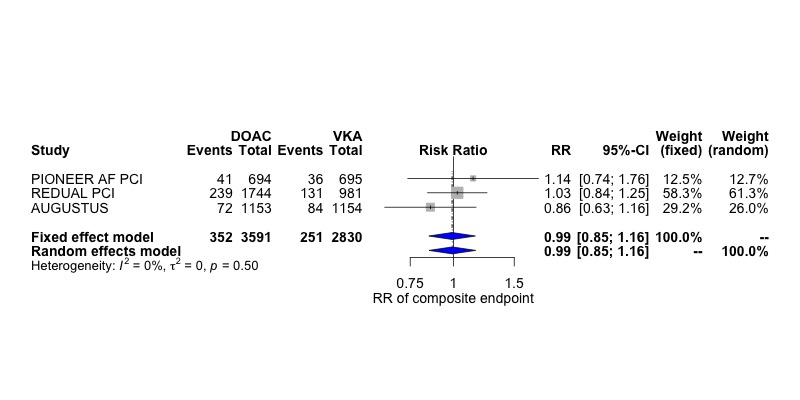
**

**Favors VKA**

**Favors DOAC**

**S2-E All-causes death**

**
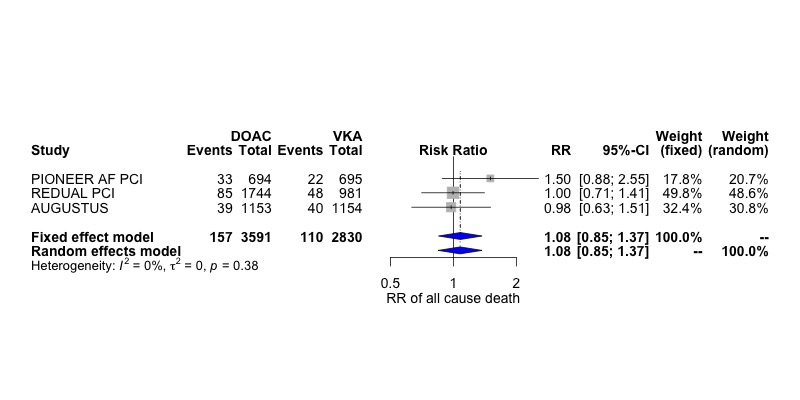
**

**Favors VKA**

**Favors DOAC**

**S2-F Stroke**

**
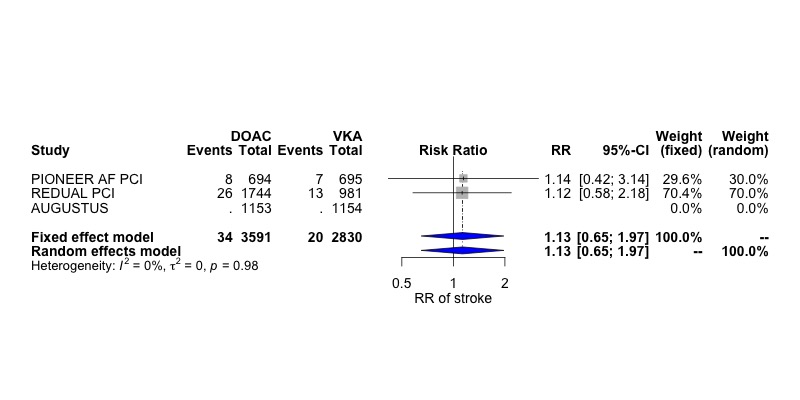
**

**Favors VKA**

**Favors DOAC**

**S2-G Myocardial infarction**

**
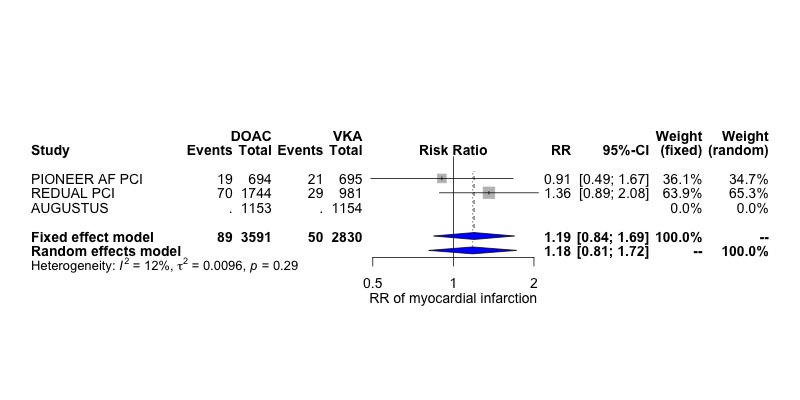
**

**Favors VKA**

**Favors DOAC**

**S2-H Stent thrombosis**

**
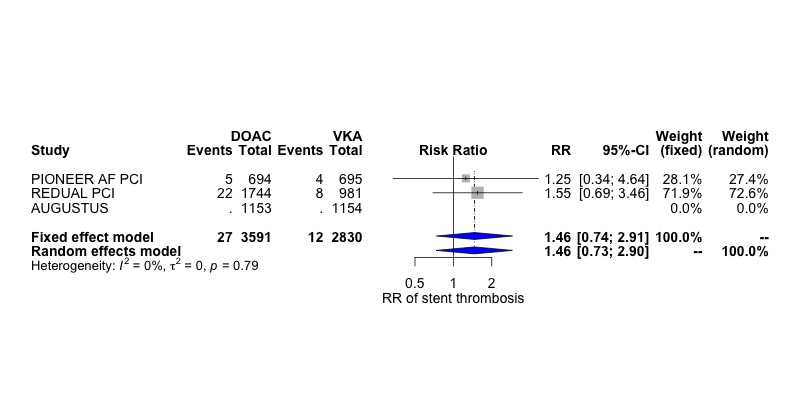
**

**Favors VKA**

**Favors DOAC**

**Figure S3: Comparison between triple therapy with DOAC and triple therapy with VKA**

**S3-A Any significant bleeding**

**
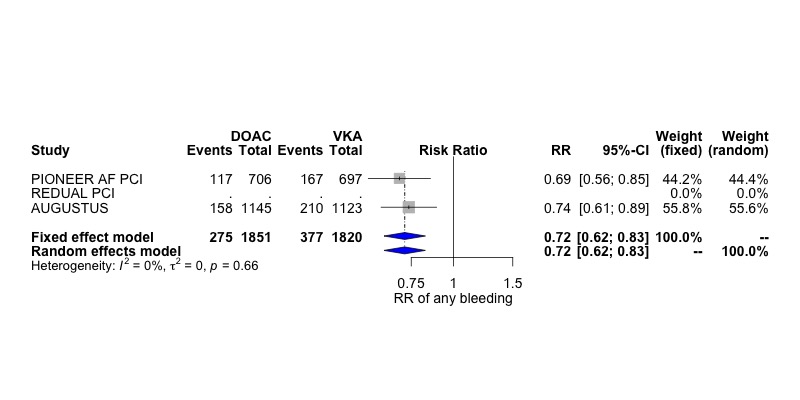
**

**Favors VKA**

**Favors DOAC**

**S3-B Major bleeding**

**
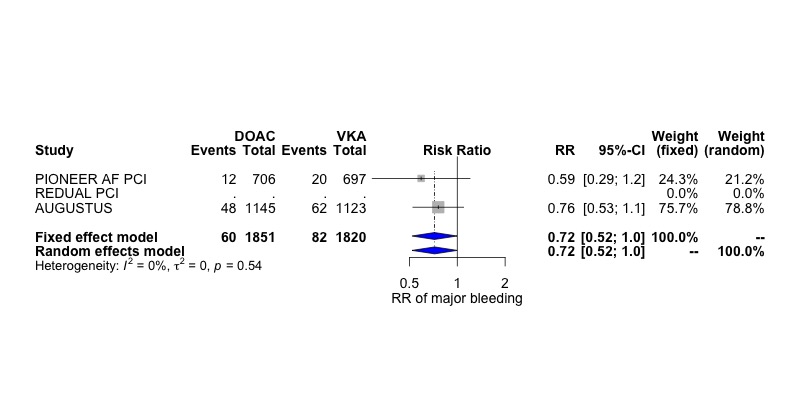
**

**Favors VKA**

**Favors DOAC**

**S3-C Composite efficacy endpoint**

**
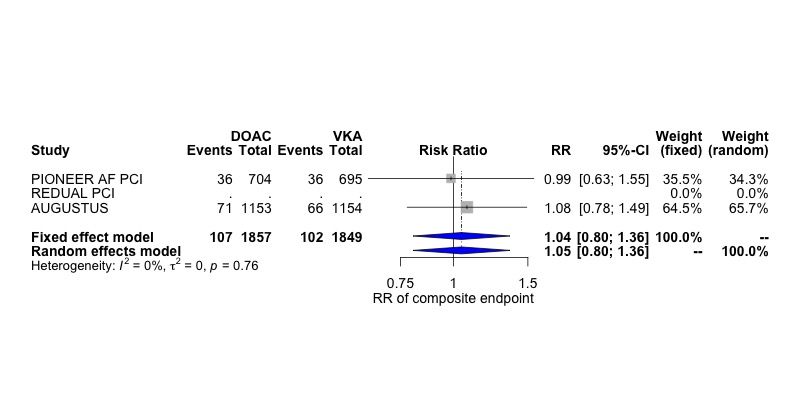
**

**Favors VKA**

**Favors DOAC**

**S3-D All-causes death**

**
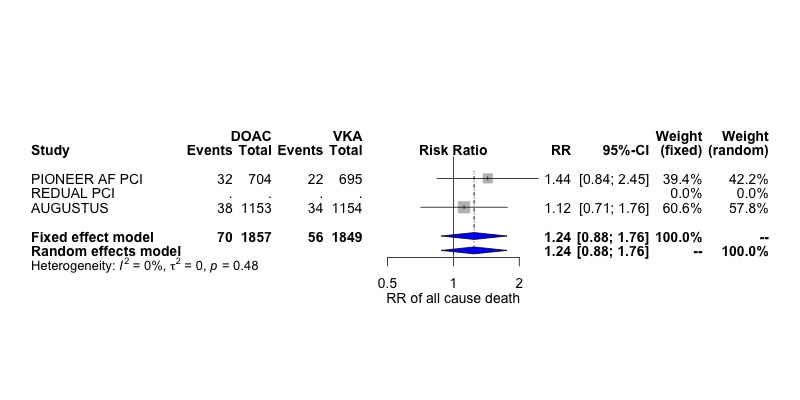
**

**Favors VKA**

**Favors DOAC**

**Figure S4: Comparison between low doses of DOAC and VKA**

**S4-A Any significant bleeding**

**
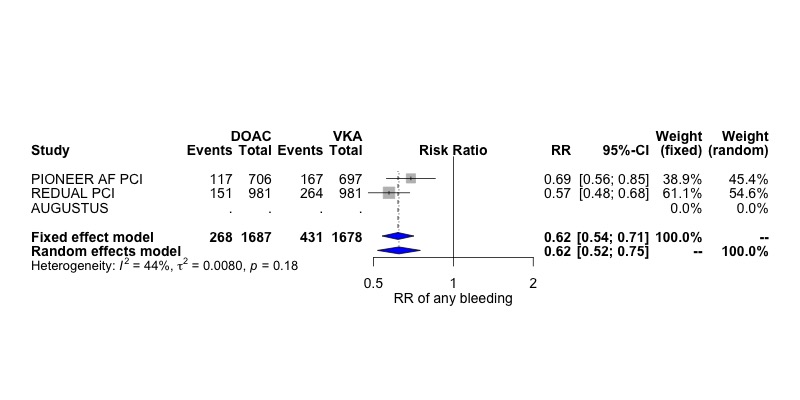
**

**Favors VKA**

**Favors DOAC**

**S4-B Major bleeding**

**
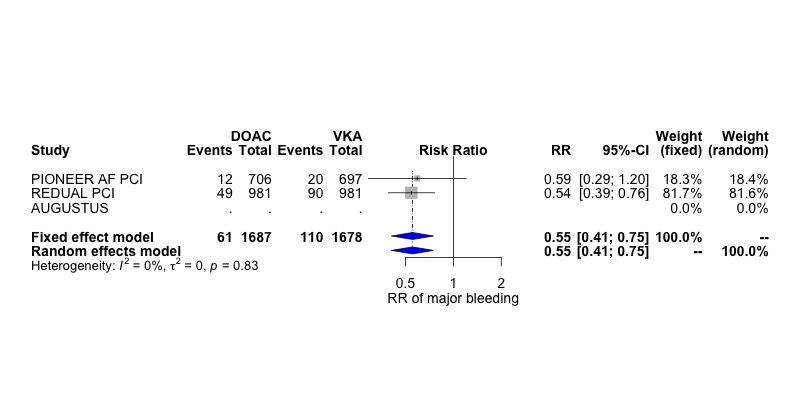
**

**Favors VKA**

**Favors DOAC**

**S4-C Intracranial bleeding**

**
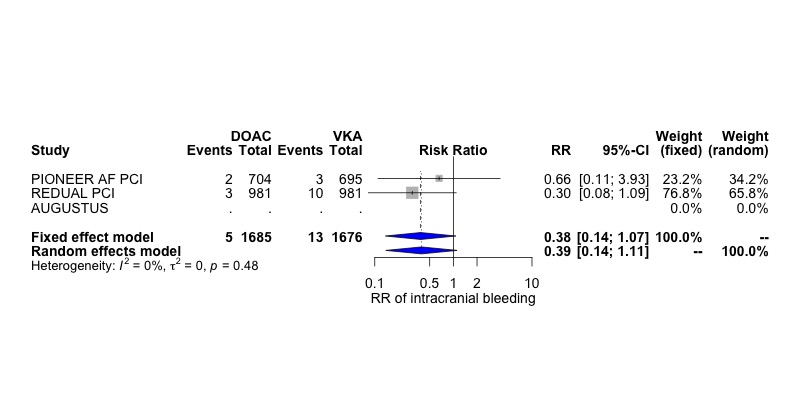
**

**Favors VKA**

**Favors DOAC**

**S4-D Composite efficacy endpoint**

**
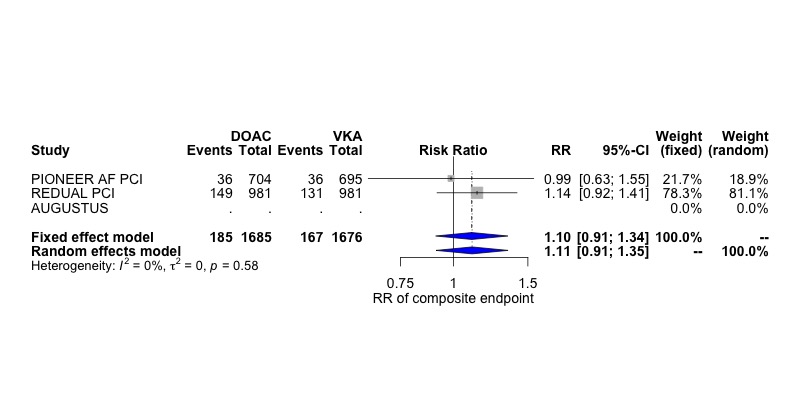
**

**Favors VKA**

**Favors DOAC**

**S4-E Stroke**

**
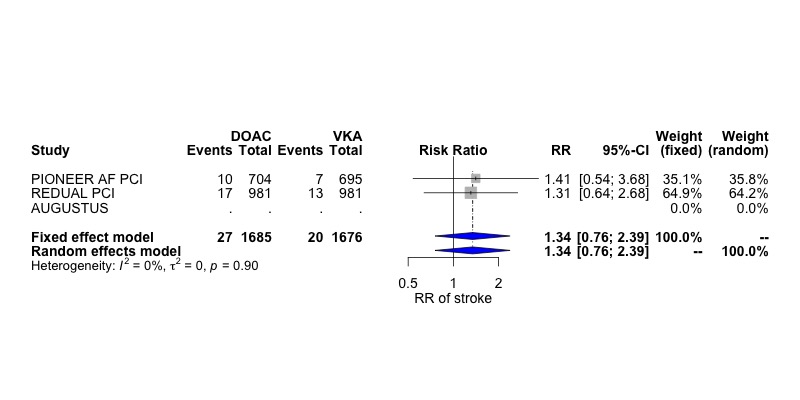
**

**Favors VKA**

**Favors DOAC**

**S4-F Myocardial infarction**

**
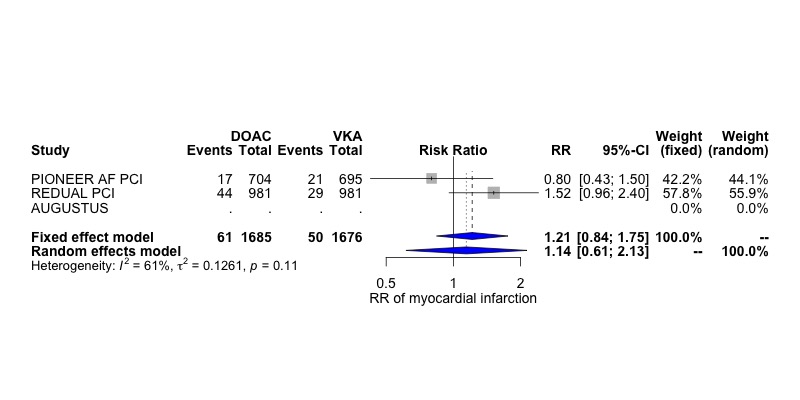
**

**Favors VKA**

**Favors DOAC**

**S4-G Stent thrombosis**

**
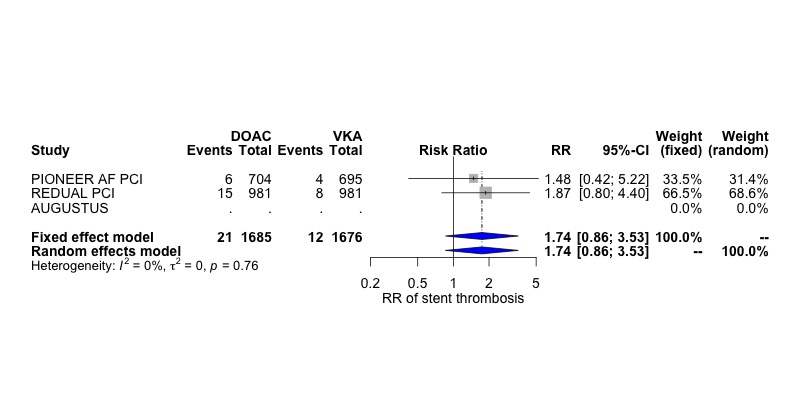
**

**Favors VKA**

**Favors DOAC**

**Figure S5: Comparison between high doses of DOAC and VKA**

**S5-A Any significant bleeding**

**
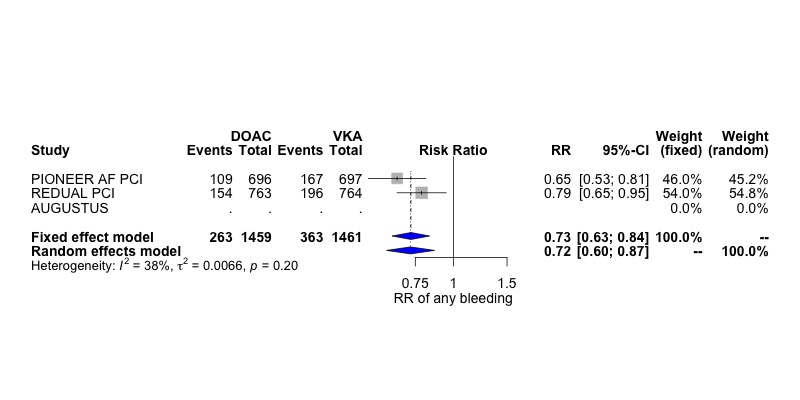
**

**Favors VKA**

**Favors DOAC**

**S5-B Major bleeding**

**
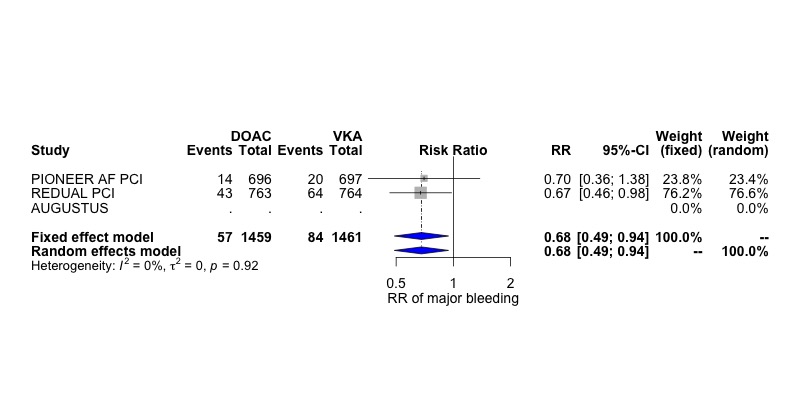
**

**Favors VKA**

**Favors DOAC**

**S5-C Intracranial bleeding**

**
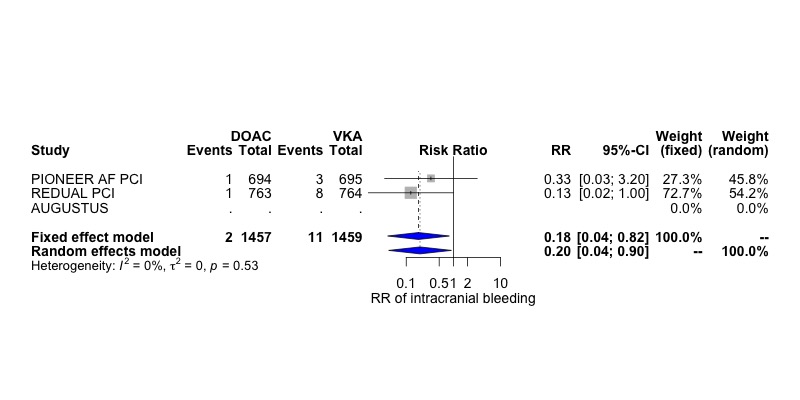
**

**Favors VKA**

**Favors DOAC**

**S5-D Composite efficacy endpoint**

**
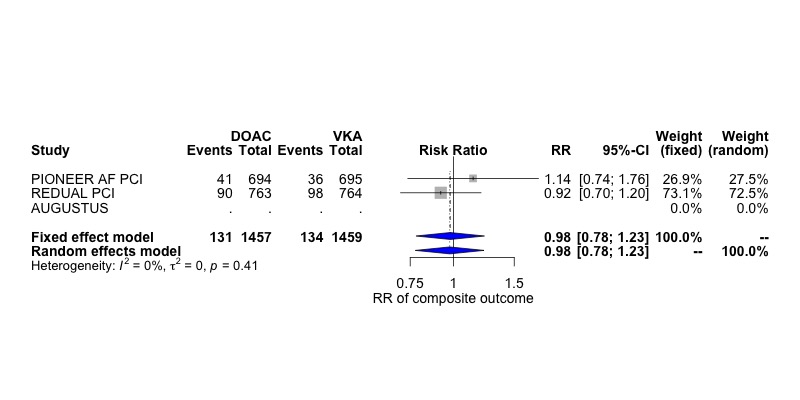
**

**Favors VKA**

**Favors DOAC**

**S5-E Stroke**

**
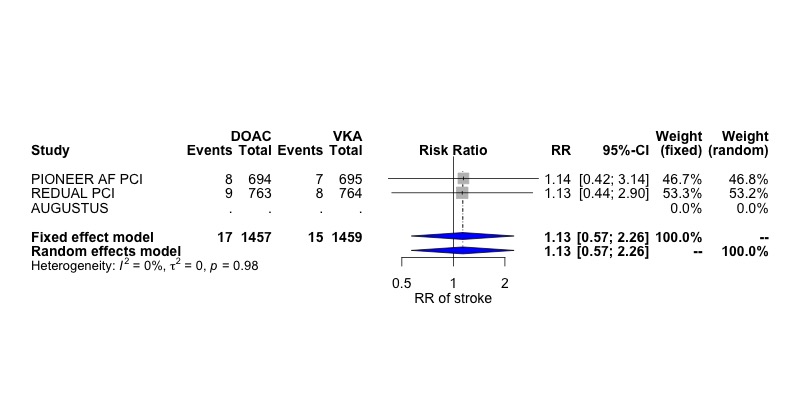
**

**Favors VKA**

**Favors DOAC**

**S5-F Myocardial infarction**

**
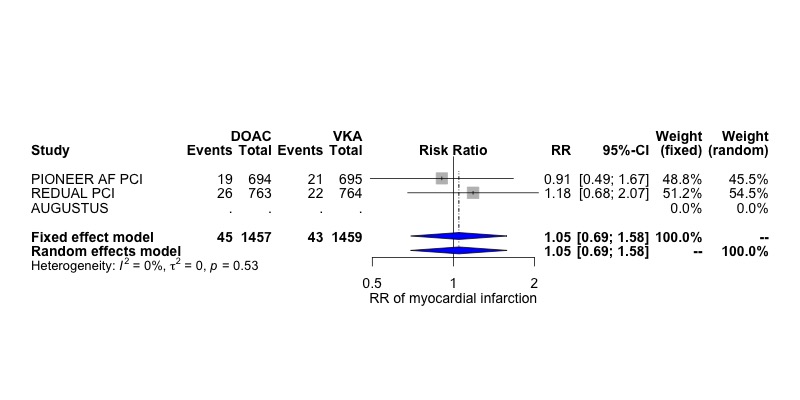
**

**Favors VKA**

**Favors DOAC**

**S5-G Stent thrombosis**

**
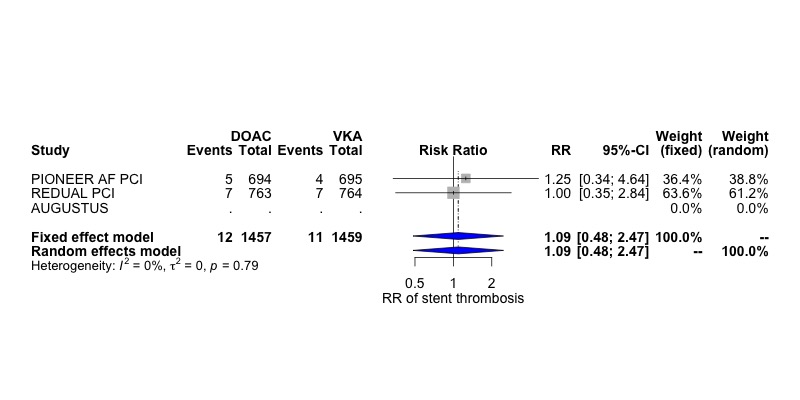
**

**Favors VKA**

**Favors DOAC**

**Figure S6: Comparison between dual therapy and triple therapy with DOAC**

**S6-A Any significant bleeding**

**
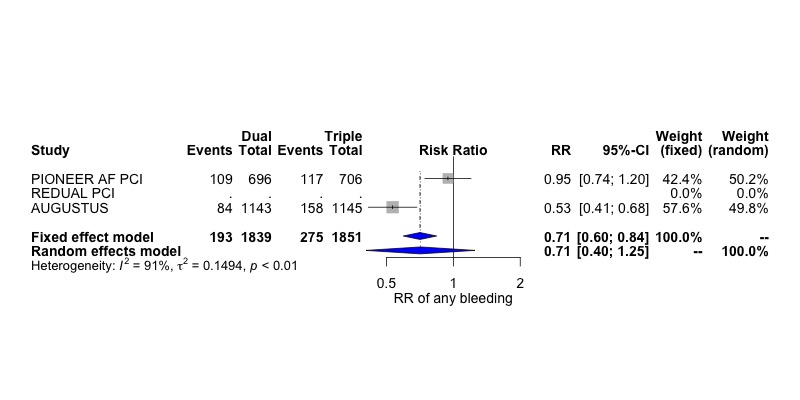
**

**Favors triple**

**Favors dual**

**S6-B Major bleeding**

**
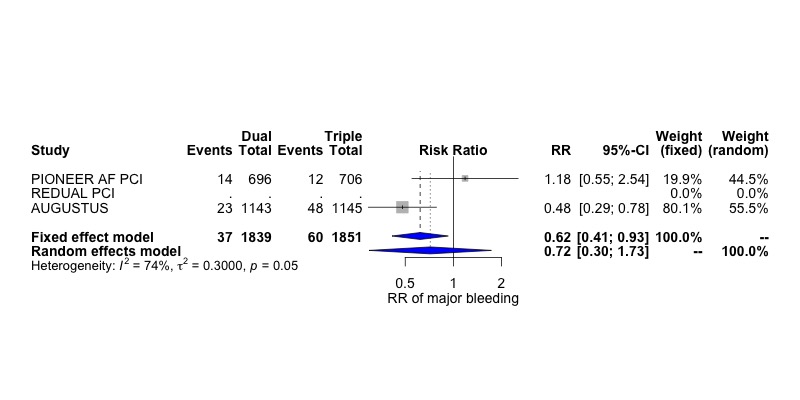
**

**Favors triple**

**Favors dual**

**S6-C Composite efficacy endpoint**

**
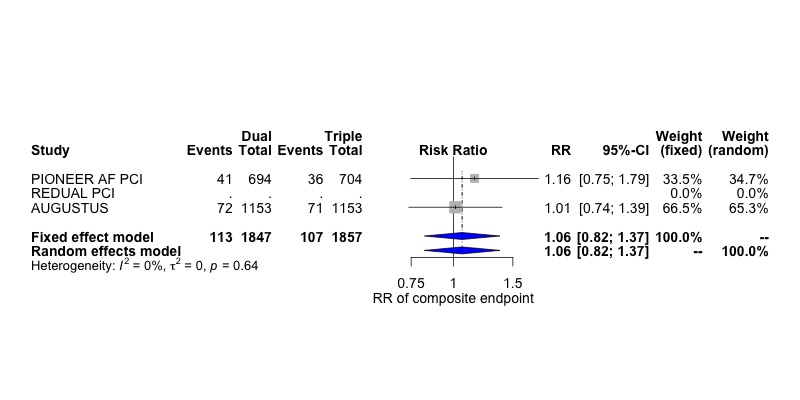
**

**Favors triple**

**Favors dual**

**S6-D All-causes death**

**
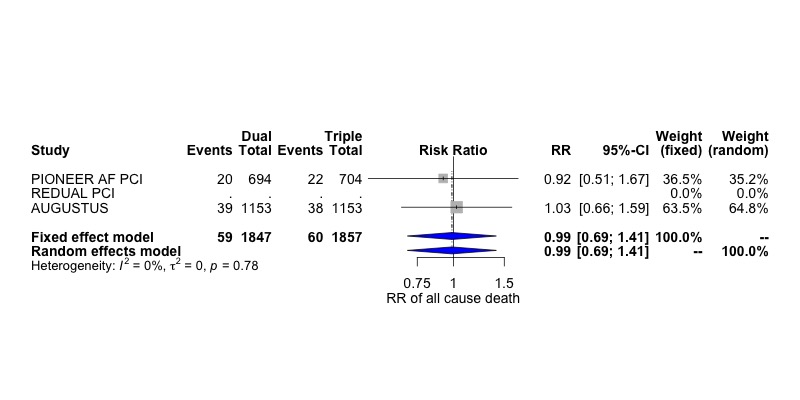
**

**Favors triple**

**Favors dual**

**S6-E Cardiovascular death**

**
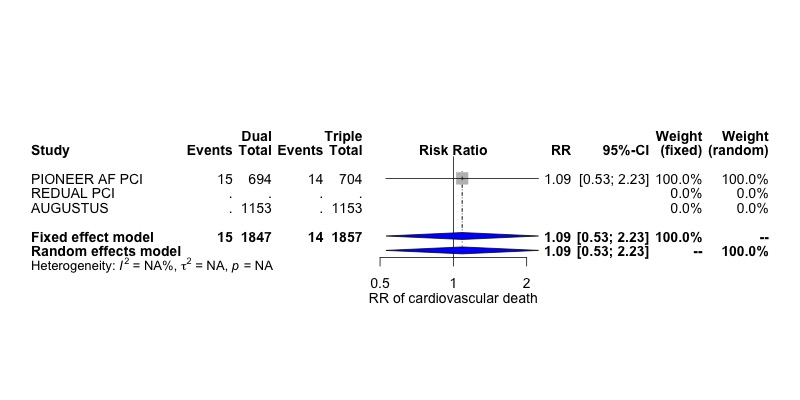
**

**Favors triple**

**Favors dual**

**Figure S7: Comparison between low dose and high dose of DOAC**

**S7-A Any significant bleeding**

**
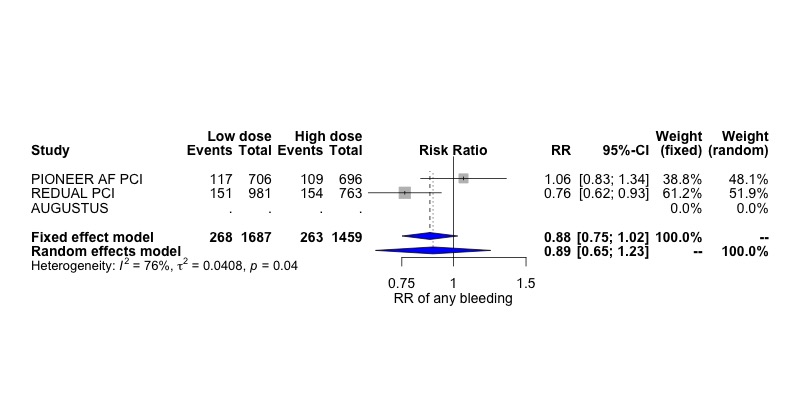
**

**Favors high**

**Favors low**

**S7-B Major bleeding**

**
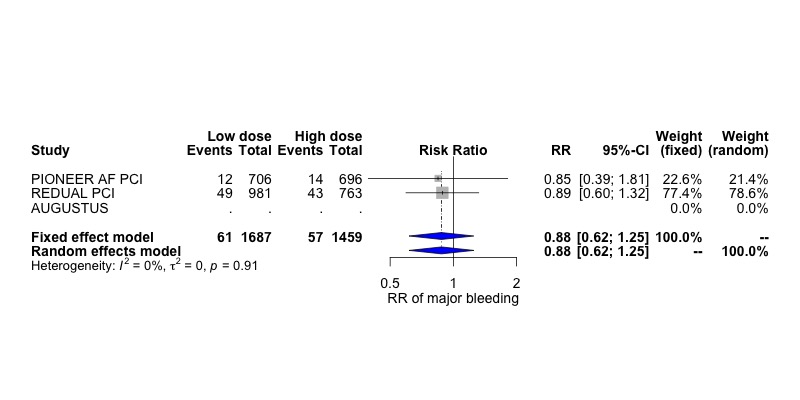
**

**Favors high**

**Favors low**

**S7-C Intracranial bleeding**

**
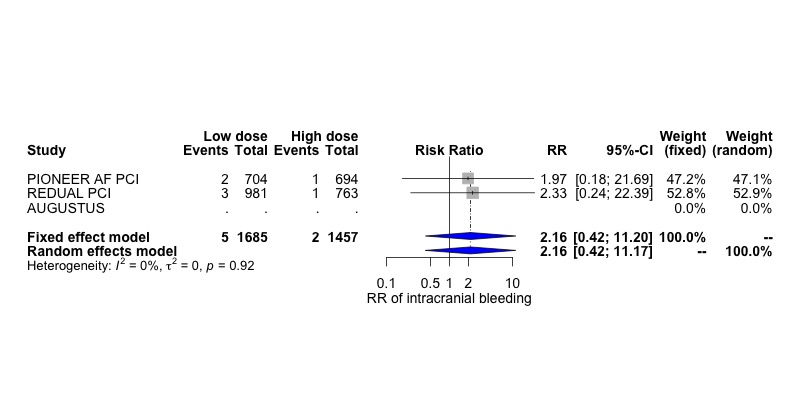
**

**Favors high**

**Favors low**

**S7-D Composite efficacy endpoint**

**
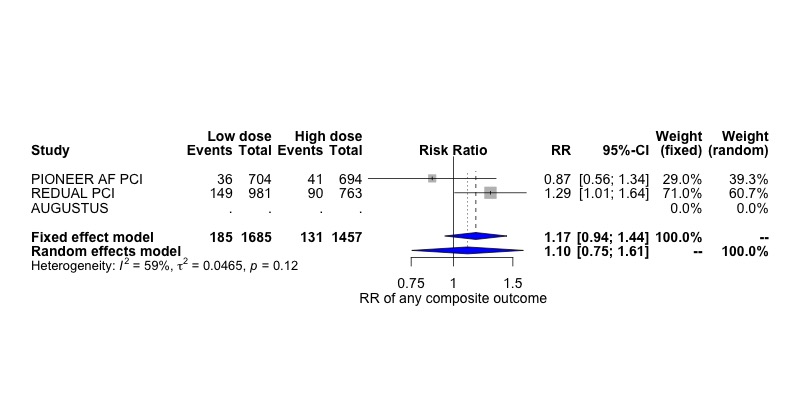
**

**Favors high**

**Favors low**

**S7-E Stroke**

**
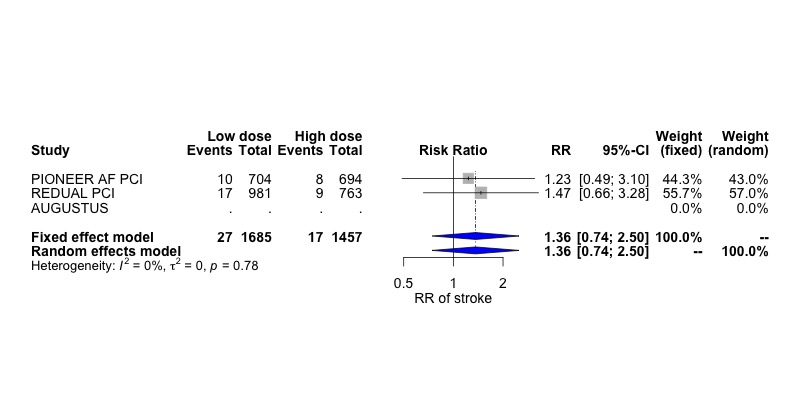
**

**Favors high**

**Favors low**

**S7-F Myocardial infarction**

**
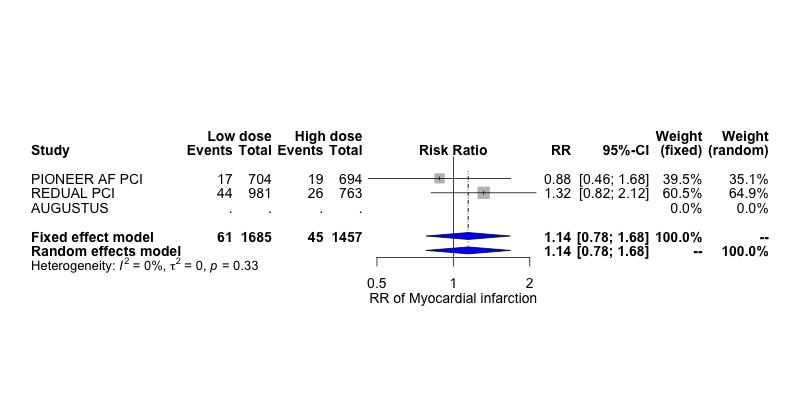
**

**Favors high**

**Favors low**

**S7-G Stent thrombosis**

**
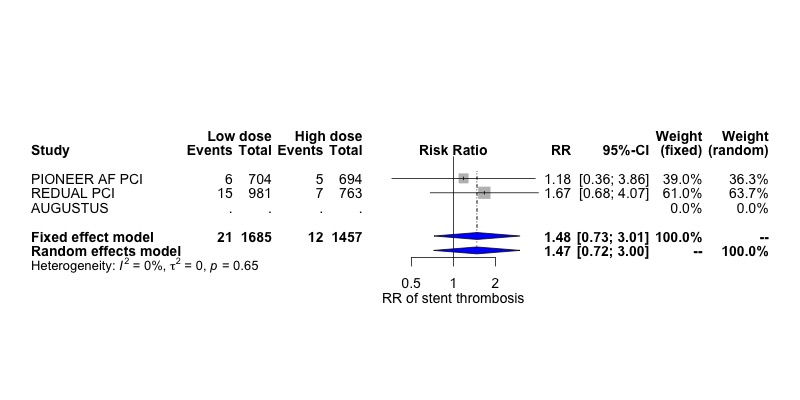
**

**Favors high**

**Favors low**

**Figure S8:** **Comparison between each DOAC subgroup (according studies) and VKA.**

PS: no subgroup data were available for AUGUSTUS trial according the dose of Apixaban.

**S8-A Any significant bleeding**

**
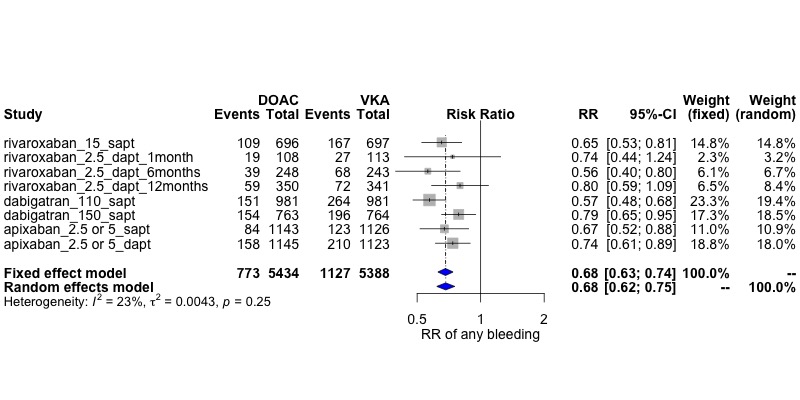
**

**Favors VKA**

**Favors DOAC**

**S8-B Major bleeding**

**
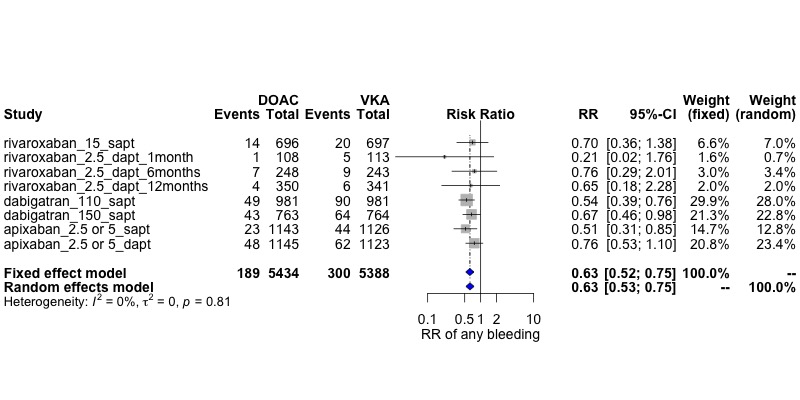
**

**Favors VKA**

**Favors DOAC**

**S8-C Composite efficacy endpoint**


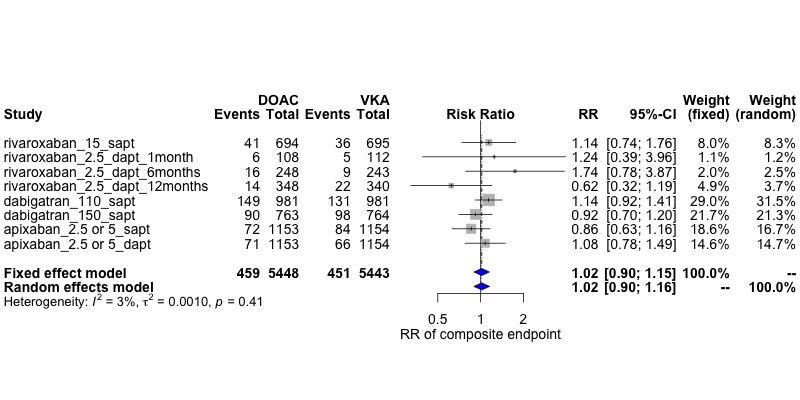


**Favors VKA**

**Favors DOAC**

**Figure S9: Funnel plots**

**S9-A Any bleeding**

**
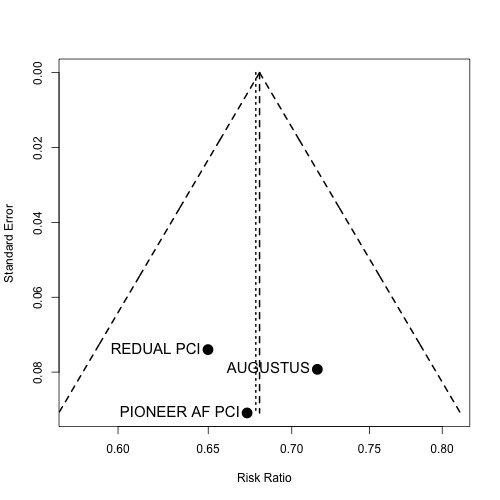
**

**S9-B Composite efficacy endpoint**

**
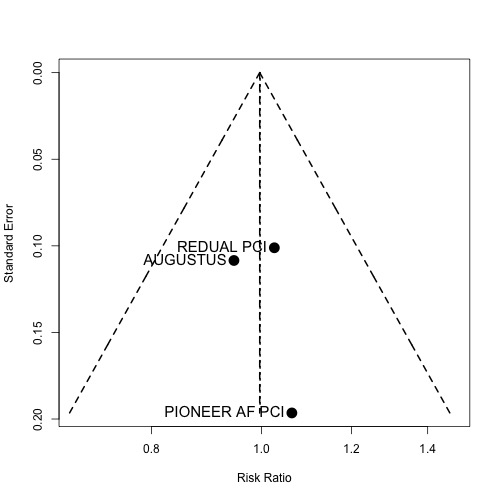
**

**Supplementary Table S1: Risk of bias in randomized studies, based on the Cochrane Risk of Bias Tool for Randomized Controlled Trials**

|  | **PIONEER AF-PCI** | **RE-DUAL PCI** | **AUGUSTUS** |
| --- | --- | --- | --- |
| Random sequence generation  *(Selection bias)* | Low risk | Low risk | Low risk |
| Allocation concealment *(Selection bias*) | Low risk | Low risk | Low risk |
| Selective reporting *(Reporting bias)* | Low risk | Low risk | Low risk |
| Blinding of participants and personnel *(Performance bias)* * | Low risk | Low risk | Low risk |
| Blinding of outcome assessment (detection bias) | Low risk | Low risk | Low risk |
| Incomplete outcome data *(Attrition bias)* | Low risk | Low risk | Low risk |
| Other sources of bias | Low risk | Low risk | Low risk |

* All trials were open label, however, since the outcome assessment was blinded (and since aspirin or matching placebo was double-blinded in AUGUSTUS), our judgement is that open label design has not influenced outcomes.

**Supplementary Table S2: Design and characteristics of the selected studies.**

| **Study** | **PIONEER AF-PCI** | **RE-DUAL PCI** | **AUGUSTUS** |
| --- | --- | --- | --- |
| **Trial type** | Randomized, open-label | Randomized, open-label | Randomized, open-label (for the regimen comparing apixaban with VKA but double-blind for the regimen comparing aspirin with placebo), 2 x 2 factorial |
| **Setting of the study** | International, multicenter | International, multicenter | International, multicenter |
| **Study enrollment** | May 2013- July 2015 | July 2014- October 2016 | September 2015- April 2018 |
| **Total number of patients** | 2124 | 2725 | 4614 |
| **Agent** | Rivaroxaban | Dabigatran | Apixaban |
| **Study arms** | - rivaroxaban 15 mg/day + SAPT  - rivaroxaban 2.5 mg twice daily + DAPT  - VKA + DAPT | - dabigatran 110 mg twice daily + SAPT  - dabigatran 150 mg twice daily + SAPT  - VKA + DAPT | - apixaban* + SAPT  -VKA + SAPT  -apixaban* + DAPT  - VKA + DAPT  (2 x 2 factorial design) |
| **Major inclusion criteria** | Age ≥ 18 years with non-valvular AF within last 1 year and who had just undergone PCI with stent placement for stable coronary artery disease or acute coronary syndrome | Age ≥ 18 years with previous non-valvular AF and who had just undergone PCI with stent placement for stable coronary artery disease or acute coronary syndrome | Age ≥ 18 years with previous non-valvular AF and recent acute coronary syndrome and/or PCI (<14 days) with a planned use of a P2Y12 inhibitor for at least 6 months |
| **Major exclusion criteria** | - History of stroke/TIA - significant gastrointestinal bleeding within 12 months before randomization  - eGFR <30 mL/min  - Hb < 10g/dl  - Any other condition known to increase the risk of bleeding | - Mechanical or biological heart valves - cardiogenic shock  - stroke, major surgery, gastrointestinal hemorrhage or major bleeding within 1 month prior to randomization  - Hb<10 g/dl  - eGFR<30 ml/min  - active liver disease | - Conditions other than AF that require chronic anticoagulation (eg, prosthetic mechanical heart valve…)  - history of intracranial hemorrhage  - eGFR <30 mL/min  - recent or planned CABG surgery for the index ACS event  - known ongoing bleeding or coagulopathies |
| **Mean follow-up** | 12 months | 14 months | 6 months |
| **Primary outcome (primary safety end-point)** | Clinically significant bleeding (a composite of TIMI major and minor bleeding, or bleeding requiring medical attention) | ISTH major or clinically relevant non-major bleeding event | ISTH major or clinically relevant non-major bleeding event |
| **Secondary outcome (secondary efficacy end-point)** | MACE (a composite of cardiovascular death, MI or stroke), each component of MACE and stent thrombosis | Composite endpoint of all-cause death, MI, stroke, systemic embolism or unplanned revascularization, each component and stent thrombosis | Composite endpoint of death, stroke, MI, stent thrombosis or urgent revascularization, composite endpoint of death and hospitalization and each component |
| AF=Atrial Fibrillation; CABG=Coronary Artery Bypass Graft; DAPT=dual antiplatelet therapy; eGFR=estimated Glomerular filtration rate; Hb= Hemoglobin; ISTH= International Society on Thrombosis and Hemostasis; MACE= Major Adverse Cardiovascular Events; MI=Myocardial Infarction; PCI=Percutaneous Coronary Intervention; SAPT=single antiplatelet therapy; TIA=Transient Ischemic Attack; TIMI= Thrombolysis In Myocardial Infarction  * 5 mg or 2.5 mg twice daily (2.5 mg if patients met two or more of the following criteria: at least 80 years of age, weight ≤ 60 kg, creatinine of at least 133 µmol per liter) | | | |
